# Effects of undernutrition on mortality and morbidity among adults living with HIV in sub-Saharan Africa: a systematic review and meta-analysis

**DOI:** 10.1186/s12879-020-05706-z

**Published:** 2021-01-04

**Authors:** Animut Alebel, Daniel Demant, Pammla Petrucka, David Sibbritt

**Affiliations:** 1 College of Health Science, Debre Markos University, Debre Markos, Ethiopia; 2 School of Public Health, Faculty of Health, University of Technology Sydney, Ultimo, NSW Australia; 3 School of Public Health and Social Work, Faculty of Health, Queensland University of Technology, Kelvin Grove, NSW Australia; 4 College of Nursing, University of Saskatchewan, Saskatoon, Canada; 5 School of Life Sciences and Bioengineering, Nelson Mandela African Institute of Science and Technology, Arusha, Tanzania

**Keywords:** Adults living with HIV, PLHIV, Sub-Saharan Africa, Undernutrition

## Abstract

**Background:**

Undernutrition is one of the most common problems among people living with HIV, contributing to premature death and the development of comorbidities within this population. In Sub-Saharan Africa (SSA), the impacts of these often inter-related conditions appear in a series of fragmented and inconclusive studies. Thus, this review examines the pooled effects of undernutrition on mortality and morbidities among adults living with HIV in SSA.

**Methods:**

A systematic literature search was conducted from PubMed, EMBASE, CINAHL, and Scopus databases. All observational studies reporting the effects of undernutrition on mortality and morbidity among adults living with HIV in SSA were included. Heterogeneity between the included studies was assessed using the Cochrane Q-test and I^2^ statistics. Publication bias was assessed using Egger’s and Begg’s tests at a 5% significance level. Finally, a random-effects meta-analysis model was employed to estimate the overall adjusted hazard ratio.

**Results:**

Of 4309 identified studies, 53 articles met the inclusion criteria and were included in this review. Of these, 40 studies were available for the meta-analysis. A meta-analysis of 23 cohort studies indicated that undernutrition significantly (AHR: 2.1, 95% CI: 1.8, 2.4) increased the risk of mortality among adults living with HIV, while severely undernourished adults living with HIV were at higher risk of death (AHR: 2.3, 95% CI: 1.9, 2.8) as compared to mildly undernourished adults living with HIV. Furthermore, the pooled estimates of ten cohort studies revealed that undernutrition significantly increased the risk of developing tuberculosis (AHR: 2.1, 95% CI: 1.6, 2.7) among adults living with HIV.

**Conclusion:**

This review found that undernutrition has significant effects on mortality and morbidity among adults living with HIV. As the degree of undernutrition became more severe, mortality rate also increased. Therefore, findings from this review may be used to update the nutritional guidelines used for the management of PLHIV by different stakeholders, especially in limited-resource settings.

**Supplementary Information:**

The online version contains supplementary material available at 10.1186/s12879-020-05706-z.

## Background

Human Immunodeficiency Virus (HIV) continues to be a significant global public health problem, with Sub-Saharan Africa (SSA) being the most significantly affected region [1, 2]. Globally, in 2018, an estimated 37.9 million people were living with HIV (PLHIV), and 1.1 million people died from Acquired Immunodeficiency Syndrome (AIDS) related illnesses worldwide [3], with 54% of PLHIV located in East and Southern Africa, 13% in Western and Central Africa, 16% in Asia and Pacific, and 6% in Western and Central Europe and North America [4]. Low and middle-income countries (LMICs), especially SSA, are the most affected region accounted for 68% of PLHIV in 2018 [2, 4]. Although there is no cure for HIV, antiretroviral therapy (ART) suppresses viral replication and increases the CD4 counts sufficiently to improve the survival rates and quality of life [5]. Despite these benefits, 23.3 million (62%) PLHIV were accessing ART in 2018 [4, 6] with low ART coverage in LMICs is mainly attributable to inaccessibility of health coverage [1].

Malnutrition refers to both undernutrition and overnutrition. Undernutrition is a state of inadequate intake of energy or nutrients to support the physiological function of the body. Due to the high prevalence of undernutrition, malnutrition often refers to undernutrition and the associated complications [7, 8]. Therefore, this review focused on undernutrition, which is the most common form of malnutrition in LMICs.

Despite the use of ART having been effective in reducing AIDS-related mortality and morbidities [9], not all patients living with HIV have the same response to therapy. Thus, additional factors, such as nutritional status, and potential negative effects on the immunologic response of PLHIV must be considered [10]. Undernutrition is the most common problem among PLHIV and a significant factor potentiating morbidities and mortality [10]. Although undernutrition and HIV are global challenges, once more, we see a higher prevalence of undernutrition in SSA [11]. Accordingly, in 2018, about 22.8% of undernourished people and 68% of PLHIV were living in SSA [2, 4, 12].

Undernutrition and HIV are found to be interwoven in a vicious cycle [13]. PLHIV are more vulnerable to developing undernutrition by different mechanisms. HIV is often accompanied by reduction in food intake due to: food insecurity, cognitive impairment or depression, medication-related nausea, and opportunistic infections (OIs) of mouth and oesophagus, which bring about painful swallowing [14]. In addition, HIV increases the energy requirements of HIV-infected adults by 10% for asymptomatic, and by 20–30% for symptomatic patients [15]. Conversely, undernutrition weakens the immune system and increases the risk of early mortality and morbidities [16, 17]. Previous studies have shown that undernutrition has a significant impact on mortality and morbidity in PLHIV [18–21], with even a minimal weight loss of up to 5% significantly increasing the risk of death [22]. Studies conducted elsewhere confirmed that low body mass index (BMI) at ART initiation hastened disease progression and increased the risk of OIs [9, 23].

To inform health program planners and policy-makers, current evidence-based findings are required. Although there is a general understanding that undernutrition and HIV are interrelated, a comprehensive systematic review and meta-analysis estimating the pooled effects of undernutrition on mortality and morbidity among adults living with HIV is lacking. Although there are primary studies reporting the effects of undernutrition on mortality and morbidity among adults living with HIV in SSA, they are highly fragmented and inconclusive. For example, some studies showed that undernutrition (BMI <  18.5 kg^2^) has a significant effect on mortality and morbidity [23–36], while others showed no significant impact on mortality and morbidity [19, 28, 37–40]. To the best of our knowledge, the above inconsistencies have not been well investigated. Therefore, this review aims to examine the effects of undernutrition on mortality and morbidities among adults living with HIV in SSA. Results obtained from this review will provide evidentiary inputs for program planners and decision-makers in designing strategies to reduce undernutrition related mortality and morbidities among PLHIV, particularly in LMICs.

## Methods

### Data sources and searching strategies

This systematic review and meta-analysis is designed to examine the effects of undernutrition on mortality and morbidity among adults living with HIV in SSA. The study protocol for this systematic review was registered in the International Prospective Register of Systematic Reviews (PROSPERO), University of York Centre for Reviews and Dissemination (ID: CRD42020161822). The Preferred Reporting Items for Systematic Reviews and Meta-Analysis (PRISMA) guideline was followed to report our results (Additional file 1) [41]. We searched articles published between 2002 and 2019, aligning with the first year in which the first WHO ART guidelines were distributed for developing countries [42]. A comprehensive search was conducted in the following databases: PubMed (contains MEDLINE), EMBASE (Elsevier), CINAHL (EBSCO), and Scopus (Additional file 2). Searches were limited to articles published in English and conducted on humans. Finally, the reference lists of included studies were screened for additional articles. Articles identified through the electronic search were exported and managed using Covidence, a primary screening and data extraction tool provided by Cochrane. The search from the above-mentioned databases was done using the following search terms:

**Line1**:“malnutrition” OR “undernutrition” OR “nutritional deficienc*” OR “malnourish*” OR “low Body Mass Index” OR “low BMI” OR “underweight” OR “nutritional status” “stunting” OR “Wasting” OR “underweight” OR “micronutrient deficienc*”.

**AND**

**Line2:** “HIV Infections” OR “HIV” OR “HIV-1” OR “HIV-2” OR “HIV infect*” OR “human immunodeficiency virus” OR “human immunedeficiency virus” OR “human immuno-deficiency virus” OR “human immune-deficiency virus” OR “((human immun*)OR (deficiency virus))” OR “acquired immunodeficiency syndrome” OR “acquired immunedeficiency syndrome” OR “acquired immunodeficiency syndrome” OR “acquired immune-deficiency syndrome” OR “((acquired immun*) OR (deficiency syndrome))” OR “HIV-positive” OR “Sexually Transmitted Diseases, Viral”.

**AND**

**Line3:** “Mortalit*” OR “incidence” OR “survival” OR “death rate” OR “risk factors” OR “time to death” OR “case fatality rate” OR “determinates” OR “mortality rate” OR “predictors” OR “opportunistic infect*” OR “AIDS related opportunistic infecti*” OR “morbidit*” OR “hospital admissions” OR “hospitalization” OR “herpes zoster” OR “bacterial pneumonia” OR “pulmonary TB” OR “extra-pulmonary TB” OR “tuberculosis” OR “TB” OR “oral candidiasis” OR “oesophageal candidiasis” OR “mouth ulcer” OR “diarrh*” OR “pneumocystis pneumonia” OR “central nervous system toxoplasmosis” OR “toxoplasmosis” OR “cryptococcal meningitis” OR “non-Hodgkins lymphoma” OR “Kaposi’s sarcoma” OR “cervical cancer” OR “herpes simplex” OR “cytomegalovirus” OR “AIDS defining disease”.

**AND**

**Line 4: “**Angola” OR “Benin” OR “Botswana” OR “Burkina Faso” OR “Burundi” OR “Cameroon” OR “Cape Verde” OR “Central African Republic” OR “Chad” OR “Comoros” OR “Republic of the Congo” OR “Democratic Republic of the Congo” OR “Cote d’Ivoire” OR “Djibouti” OR “Equatorial Guinea” OR “Eritrea” OR Ethiopia” OR “Gabon” OR “The Gambia” OR “Ghana” OR “Guinea” OR “Guinea-Bissau” OR “Kenya” OR “Liberia” OR “Madagascar” OR “Malawi” OR “Mali” OR “Mauritania” OR “Mauritius” OR “Mozambique” OR “Namibia” OR “Niger” OR “Nigeria” OR “Rwanda” OR “Sao Tome and Principe” OR “Senegal” OR “Seychelles” OR “Sierra Leone” OR “Somalia” OR “South Africa” OR “South Sudan” OR “Sudan” OR “Swaziland” OR “Tanzania” OR “Togo” OR “Uganda” OR “Zambia” OR “Zimbabwe”.

The PICO framework was used to determine the eligibility for the study:

**✓ P**articipants/population: adults (defined as those aged ≥15 years) living with HIV.

**✓ I**ntervention(s)/exposure(s) group: undernourished adults living with HIV.

**✓ C**omparator(s)/control group: well-nourished adults living with HIV.

**✓ O**utcomes of interests: mortality and morbidities among adults living with HIV.

### Inclusion and exclusion criteria

**Fig. 1:**
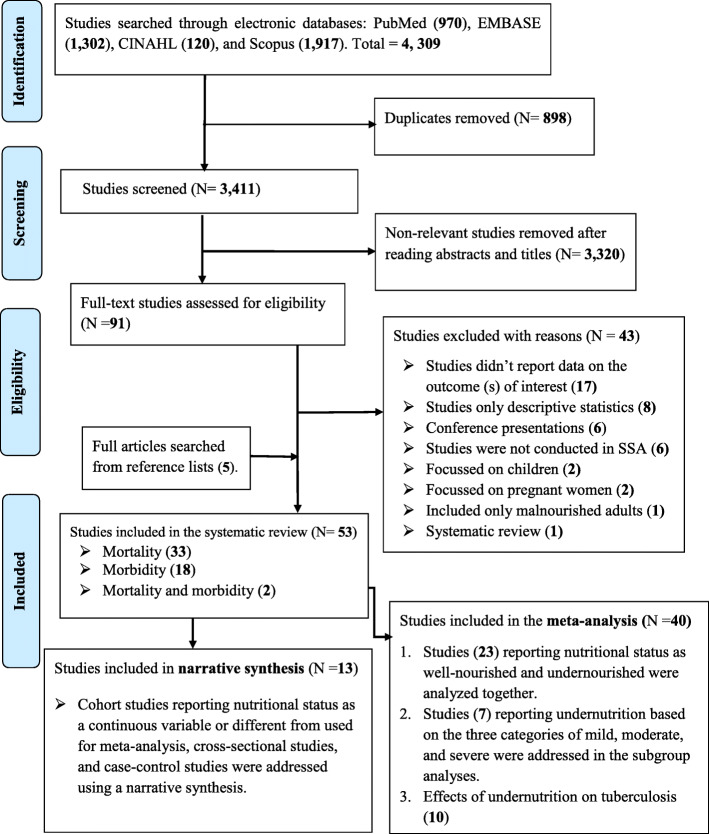
Flow chart of study selection for a systematic review and meta-analysis of the effects of undernutrition on mortality and morbidity among adults living with HIV in SSA

The study selection was done by the primary author (AA) using a two-stage approach. Initially, studies were screened based on titles and abstracts. At this stage, all studies reporting mortality and morbidity among PLHIV were considered. Then, a full-text assessment based on the predetermined inclusion criteria was performed (Fig. 1). All observational studies (i.e., cross-sectional, case-control, and cohort) reporting effects of undernutrition on mortality and morbidly among adults living with HIV in SSA were considered for inclusion. However, only cohort studies reporting the adjusted hazard ratio were included in the meta-analysis as determination of cause and effect relationships requires a robust study design. Excluded were systematic reviews, animal studies, studies not reporting the outcome of interests, conference papers, and editorial comments. The reason for excluding conference papers was due to the inability to assess the quality of studies in the absence of their full texts. Furthermore, studies conducted among HIV-infected pregnant women were excluded as pregnancy by itself increased the risk of undernutrition, and nutritional assessment tools used for pregnant women are different from tools used for other adults [43]. Studies involving both HIV-infected and HIV-uninfected adults were excluded, unless data for HIV-infected adults were reported separately. Articles included only malnourished adults living with HIV were also not considered for this review as these lacked controls (i.e., well-nourished adults living with HIV).

### Measurement of outcome variables

This systematic review focused on two outcomes. The first outcome was the effect of undernutrition on mortality among adults living with HIV. Undernutrition (underweight) was defined as a BMI of less than 18.5 kg/m^2^. The severity of undernutrition was classified as severe (BMI < 16 kg/m^2^), moderate (BMI 16–16.99 kg/m^2^), and mild (BMI 17–18.48 kg/m^2^) [44]. The second outcome was the effect of undernutrition on morbidities. Morbidity refers to the occurrence of any type of opportunistic infection, incidence of AIDS defined diseases, hospital admissions, and other types of illnesses related to HIV-infection as reported by each primary study. The pooled effects of undernutrition on mortality and morbidity were determined using the (adjusted) hazard ratios reported from primary studies. For the meta-analysis, only cohort studies reporting the adjusted hazard ratios were included, as described previously.

### Quality appraisal

The quality of included studies was appraised using the Newcastle-Ottawa scale (NOS) risk-of-bias assessment tool for cross-sectional, cohort, and case-control studies [45]. The NOS is validated for case-control and cohort studies with grading from zero to ten for cross-sectional, and zero to nine for case-control and cohort studies [46]. The three components of the tool are: selection, comparability, and outcome/exposure. The selection part of this tool was graded from zero to five stars for cross-sectional studies, and zero to four stars for cohort and case-control studies. The comparability was graded from zero to two stars for all study designs. Lastly, the outcome/exposure was primarily related to the statistical analysis and cofounding handling mechanisms, which was graded from zero to three stars for all study designs.

During the quality appraisal, three (AA, DD, and DS) authors were involved, ensuring each study was appraised by two authors, with any disagreements between authors resolved through discussion. Finally, the quality score of each study was calculated as the sum of scores, thus ranging from zero to ten for cross-sectional studies, and zero to nine for cohort and case-control studies. Accordingly, articles receiving three or four stars in the selection, one or two stars in comparability, and two or three stars in outcomes were categorized as “good quality”. Articles with two stars in the selection, one or two stars in comparability, and two or three stars in outcomes, were classified as “fair quality”, whereas, a “poor quality” score was considered if the articles got zero or one-star(s) in the selection, or zero stars in comparability, or zero or one-star(s) in outcomes [47].

### Data extraction

A standardized data extraction format was adopted and prepared based on the Joanna Briggs Institute (JBI) data extraction format [48]. The data extracted included the following: primary author, publication year, country/countries where the study was conducted, study design, study/follow-up period, sample size, sex/ gender of participants, mortality rate/morbidity rate, and adjusted hazard ratio/ adjusted odds ratio with 95% confidence intervals (CI). If further information or clarification was needed, the primary author of the original article was contacted through email. The article was excluded if, after at least two email attempts, the author did not respond. Before combining in a meta-analysis, the hazard ratios were transformed into a logarithmic scale as the hazard ratio was measured on a ratio (exponential) scale.

### Data synthesis

A narrative synthesis approach was employed to present results, which were not included in the meta-analysis. A meta-analysis was performed using Stata™ Version 16 statistical software to estimate effect sizes. Effect sizes were expressed as log-adjusted hazard ratios (AHR) with their 95% CIs. In this meta-analysis, well-nourished adults living with HIV were considered as a reference (control) category. However, some primary studies reported AHR by considering undernourished adults living with HIV as a reference category, so to ensure consistency and uniformity, new AHRs with their 95% CIs were calculated by taking the reciprocal of the reported AHRs [49]. Primary studies reporting the AHR based on the severity of undernutrition were included in our subgroup analysis because they reported nutritional status in three (mild, moderate, and severe) categories rather than two categories (undernutrition versus well-nourished). Studies reporting nutritional status (BMI) as a continuous variable were addressed in the narrative review.

### Heterogeneity, publication bias, and subgroup analyses

The presence of heterogeneity between included studies was assessed using Cochrane Q-test and I^2^ statistics. The I^2^ value can be interpreted as: 0 to 40% (might not be important); 30 to 60% (may represent moderate heterogeneity); 50 to 90% (may represent substantial heterogeneity); and 75 to 100% (considerable heterogeneity) [50]. In the case of significant heterogeneity, possible sources were investigated by performing univariate meta-regression analyses, and a random-effects meta-analysis model estimated the final effect size. Furthermore, to minimize random variations between primary studies, subgroup analyses were performed based on different variables (i.e., country where studies were conducted, degree of malnutrition, sample size, publication year, and quality score). We selected these variables because of the availability of data for these variables from most included studies. At last, the presence of publication bias was assessed using Egger’s and Begg’s tests at a 5% significance level [51].

## Results

### Identification of studies

A total of 4309 articles were identified from PubMed, EMBASE, Scopus, and CINAHL (Fig. 1). After the removal of 898 duplicates, 3411 studies remained and were screened for title and abstract. In the next step, 3320 articles were excluded based on titles and abstracts as these were not relevant for this review. The full text of 91 studies were downloaded and assessed based on the predefined inclusion criteria. An additional 43 full texts were excluded for the following reasons: 17 studies did not report data on the outcome(s) of interest [52–65], eight studies reported only descriptive results [66–76], six studies were conference presentations [77–82], six studies were not conducted in SSA [83–88], two studies focused on children (aged < 15 years) [89, 90], two studies focused on pregnant women [91, 92], one study included only malnourished adults [93], and one was a review paper [94]. Five articles were added from the reference lists of included articles, leading to 53 included studies. Of these, 33 studies were conducted on mortality, 18 studies on morbidity, and two on both morbidity and both morbidity. Finally, 40 studies were available for the meta-analysis. Of these, 23 studies were used to estimate the pooled effects of undernutrition on mortality. Seven studies were included in the subgroup analysis to determine the effects of the severity of undernutrition (mild, moderate, and severe) on mortality. The remaining ten studies were used to estimate the pooled effects of undernutrition on tuberculosis.

### Description of included studies

**Fig. 2:**
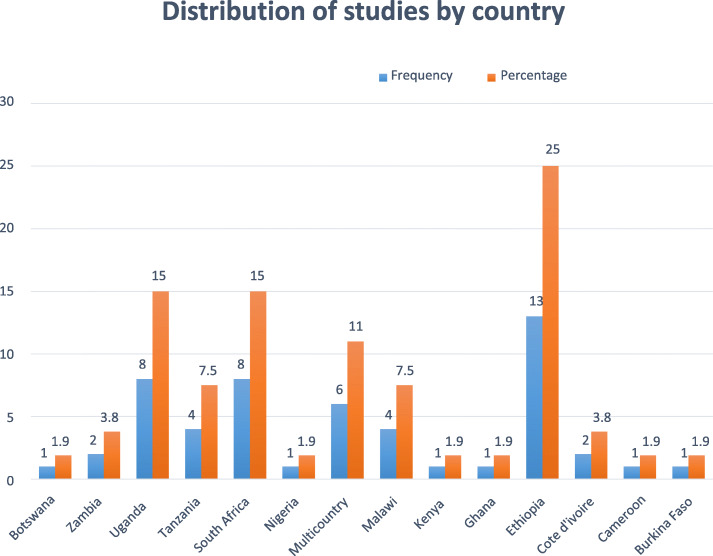
The distribution of included studies across countries in SSA

In this systematic review, a total of 367,680 adults living with HIV were included across included articles with more than three quarters (76.2%) being females. Publication year of the included studies ranged from 2006 to 2019. The sample size of the included studies ranged from 71 in Kenya [95] to 68,378 in Tanzania [96]. Most were cohort studies (*n* = 47, 88.7%). From the 32 studies that reported the number of deaths or proportion of mortality, the highest (38%) mortality was reported from a study done in Kenya [95]; whereas the lowest (2%) proportion mortality was reported from a study done in Cote d’Ivoire [25]. From the 19 included studies to assess the effects of undernutrition on morbidities, 14 studies were done on TB, two studies on anaemia, and one study each for intestinal parasite (IP), AIDS-defining disease, and OIs. Of the18 studies that reported the number of morbidities or proportion of morbidity, the highest proportion of morbidity (65%) was recorded in a study conducted in Uganda [97]. Conversely, the lowest (2.4%) proportion of morbidity was reported from a study conducted in South Africa [32] (Table 1). In this review, 13 SSA countries and six multi-country based studies were represented. In this regard, more than half (55%) of the studies were conducted in Uganda (*n* = 8), Ethiopia (*n* = 13), and South Africa (*n* = 8) (Fig. 2).

**Table 1:** Descriptive summary of 53 included studies in the systematic review of the effects of undernutrition on mortality and morbidity among adults living with HIV, between 2002 and 2019

	**Studies included assessing the effect of undernutrition on mortality**								
**No**	**Author** **(Publication Year)**	**Study Design**		**Study/ follow-up period**	**Quality score**	**Sample size**	**Female N (%)**	**Mortality N (%)**	**Adjusted confounders for mortality**
1.	Ferradini et al., (2006) [24]	Retrospective cohort		2001–2004	5	1, 308	827 (63.2)	243 (19)	Age, year of HAART initiation, type of ART regimen, follow-up site, sex, and baseline CD4 count
2.	Evans et al., (2012) [29]	Retrospective cohort		2004–2009	8	8409	5204 (61.9)	661 (7.9)	Hgb, CD4 count, aspartate transaminase, TB, age, and sex
3.	Palombi et al., (2009) [98]	Retrospective cohort		NR	6	3, 749	2325 (62)	393 (10.5)	Sex, age, baseline Hgb, baseline CD4 count, baseline HIV RNA level, WHO staging, ART adherence, and length of ART follow-up.
4.	Jerene et al., (2006) [99]	Cohort		2003–2005	6	152	66 (43.4)	24 (15.8)	TLC, WHO staging and Hgb
5.	Johannessen et al., (2008) [100]	Prospective cohort		2003–2006	7	320	223 (69.7)	95 (29.7)	Sex, WHO staging, ART start year, Hgb, TLC, and platelet count
6.	Moh et al., (2007) [25]	Cohort		2002–2004	7	792	606 (76.5)	18 (2)	WHO staging, Hgb, viral load, baseline CD4 count, follow-up CD4 count and follow-up viral load
7.	Brown et al., (2016) [39]	Cohort		2008–2015	8	432	243 (56.3)	74 (17.2)	Sex, age at enrolment, CD4 count at enrolment, year of enrolment, and route of HIV testing
8.	Tesfamariam et al., (2016) [18]	Retrospective cohort		2006–2013	8	489	254 (51.9)	87 (17.8)	Educational status, functional status, WHO staging, CD4 count, Hgb, previous OI, HIV related counselling
9.	Bastard et al., (2013) [31]	Cohort		2004–2010	8	55,789	36,508 (65.4)	1843 (3.3)	Sex, age, WHO staging, CD4 count, diagnosis of TB, eligibility to ART, and mode of entry to ART
10.	Dao et al., (2011) [101]	Prospective cohort		2005–2007	7	661	661 (100)	53 (8)	Country, age, WHO staging, diagnosis of TB, CD4 count, viral load, prophylaxis, Hgb, WBC, neutrophils count, platelet counts, potassium, chloride, sodium, hyponatremia, creatinine clearance, AST/ALT, and albumin
11.	Kouanda et al., (2012) [30]	Retrospective cohort		2003–2008	8	5608	3926 (70)	690 (12.3)	Age, sex, occupation, WHO staging, ART regimen, CD4 count, year of HAART initiation, and intensity of intervention
12.	Masiira et al., (2014) [102]	Prospective cohort		1992–2011	8	374	204 (54.5)	27 (7.21)	Age, sex, marital status, alcohol consumption, tobacco use, Hgb, CD4 count, WHO staging, viral load, malaria infection during follow-up, diarrhea, and viral load
13.	Teshome et al., (2015) [38]	Retrospective cohort		2011–2012	6	1173	649 (55.3)	47 (4)	Sex, gap b/n test and treatment, marital status, family size, facility type, CD4 count, age, INH prophylaxis, CPT prophylaxis, side effects, functional status, HIV status disclosure, educational status, TB, and WHO staging
14	Chen et al., (2008) [103]	Retrospective cohort		2004–2006	7	2, 838	1716 (60.5)	376 (13.2)	Sex, age, and WHO staging
15.	Sieleunou et al., (2009) [104]	Retrospective cohort		2001–2006	7	1, 187	660 (55.6)	338 (28.5)	Sex, WHO staging, CD4 count, and Hgb
16.	Liu et al., (2011) [20]	Retrospective cohort		2004–2009	7	8271	11,927 (65.3)	1673 (9.2)	Hgb and MUAC
17.	Ssebutinde et al., (2018) [35]	Retrospective cohort		2006–2012	7	8364	5308 (63.5)	180 (2.1)	Age, sex, WHO staging, CD4 count, and level of education
18.	Maskew et al., (2013)	Retrospective cohort		2004–2010	8	7354	4621 (62.8)	333 (4.5)	Sex, age, CD4 count, and Hgb
19.	Geng et al., (2013) [105]	Retrospective cohort		2007–2011	7	2633	1563 (59.4)	42 (1.6)	Age, sex, CD4 count, baseline TB diagnosis, pregnancy at ART initiation, WHO staging, income, employment status, education, and distance from ART clinic
20.	Hoffmann et al., (2011) [106]	Retrospective cohort		2003–2008	7	15,060	5455 (36.2)	2658 (18)	Sex, age, WHO staging, TB symptoms, Hgb, viral load, and CPT
21.	Toure et al., (2008) [27]	Retrospective cohort		2004–2007	6	10,211	7187 (70.4)	1140 (11)	Sex, age, CD4 count, WHO staging, ART regimen, Type of HIV, and Hgb
22.	Damtew et al., (2015) [21]	Retrospective cohort		2007–2011	6	784	485 (61.9)	87 (11.1)	Marital status, educational status, functional status, WHO staging, CD4 count, anaemia, and TB co-infected
23.	Ayele et al., (2017) [23]	Retrospective cohort		2012–2014	7	280	183 (65.4)	NR	Sex, age, educational level, residence, religion, occupation, marital status, alcohol, WHO staging, CD4 count, TB treatment, type of ART regimen, and prophylaxis
24.	Maman et al., (2012) [107]	Retrospective cohort		2001–2010	7	24, 037	16,355 (68)	568 (2.4)	HIV program, sex, age, WHO staging, initial CD4 count, updated CD4 count, and year of ART initiation
25.	Naidoo et al., (2018) [34]	Retrospective cohort		2008–2010	6	948	547 (57.7)	56 (5.9)	Age, sex, CD4 count, WHO staging, and TB
26.	Stringer et al., (2006) [108]	Cohort		2004–2005	7	21,755	13,646 (62.7)	1, 120 (5.1)	Age, ART non-adherence, sex, Hgb, CD4 count, WHO staging, and TB infection
27.	Hussen et al.,(2016) [19]	Retrospective cohort		2006–2011	8	340	200 (58.8)	42 (12.4)	Age, marital status, CD4 count, WHO staging, occupation, educational level, Fluconazole prophylaxis, and Baseline HAART
28.	Pac et al., (2015) [109]	Prospective cohort		2010–2012	7	540	324 (60)	39 (7.2)	Age, sex, CD4 count, Hgb, TB infections, and positive serum Cr Ag
29.	Tchounga et al., (2016) [110]	Cohort		2014–2015	7	1825	1102 (60.4)	221 (12.1)	Sex, age, year of ART initiation, WHO staging, CD4 count, and Hgb
30.	Nansera et al., (2012) [37]	Retrospective cohort		2007–2010.	6	386	142 (36.8)	53 (13.7)	Sex, anaemia, CD4 cell count, and WHO staging
31.	Otwombe et al., (2014) [111]	Prospective cohort		2003–2010	8	2221	1555 (70)	242 (11)	Sex, time on ART, CD4 count, employment status, ever smoking, and ever TB
		Prospective cohort		2003–2010		4469	3480 (77.9)	324 (7.2)	
32.	Kendi et al., (2013) [95]	Retrospective cohort		2005–2009	6	71	35 (49.3)	27 (38)	Age, sex, CD4 count, on ART, and any anti-fungal therapy
33	Zachariah et al., (2006) [112]	Cross-sectional		2003–2005	9	1, 507	990 (65.7)	190 (12.6)	Sex, age, WHO staging, CD4 count, and active TB
34	Umanah et al., 2015) [18]	Cross-sectional		2007–2010	8	947	490 (51.7)	NR	ART regimen, age, sex, site of TB, CPT prophylaxis, CD4 categories, Hgb, infiltrative cavitation change on X-ray, fibrotic change on X-ray, other OIs, comorbidity, and adverse events to medication
	**Studies included assessing the effect of undernutrition on morbidity**								
**No**	**Author**	**Study Design**		**Study/ follow-up period**		**Sample size**	**Female** **N (%)**	**Morbidity N (%)**	**Adjusted for confounders for morbidities**
35.	Moore et al., (2007) [26]	Prospective cohort		2003–2005	6	1042	765 (73.4)	53 (5.1)	Sex, median age, CD4 count, viral load, prior TB treatment, participation in previous safe water/co-trimoxazole study
36.	Kufa et al., (2016) [32]	Prospective cohort		2011–2012	7	634	513 (80.9)	15 (2.4)	Sex, age, employed, ever smoked, alcohol drinking, duration since HIV test, on ART, current or prior ART use, current CPT use, previous TB treatment, and CD4 count
37.	Sabasaba et al., (2019) [96]	Retrospective cohort		2011–2014	8	68,378	51,486 (75.3)	3124 (4.6)	Age, sex, marital status, CD4 categories, WHO staging, CPT use, IPT status, ART status, and functional status at enrolment
38.	Worodria et al., (2011) [113]	Prospective cohort		NR	7	219	157 (71.7)	6 (3.8)	Age, Sex, TB ski test, C-reactive protein, Hgb, CD4 count, and WHO staging
39.	Ahmed et al., (2018) [33]	Retrospective cohort		2010–2015	8	451	267 (59.2)	119 (26.4)	Marital status, family size, substance use, previous TB, OIs, bed ridden, length of follow-up, WHO staging, Hgb, CD4 count, and IPT
40.	Tiruneh et al., (2019) [36]	Retrospective cohort		2009–2012	8	600	356 (59.3)	53 (8.8)	Age, CD4 count, sex, weight, WHO staging, functional status, CPT, previous TB treatment, and OI
41	Nicholas et al., (2011) [114]	Prospective cohort		2006–2008	7	28,323	18,968 (67.0)	780 (9) pre 933 (5) pos	Residence, sex, age, history of ART use, TB history, and CD4 count
42.	Liu et al., (2015) [115]	Prospective cohort		2004–2012	8	67,686	50,633 (74.8)	7602 (11.2)	Age, sex, district, family size, years of enrolment, season of visit, MUAC, anaemia, CD4 count, WHO staging, ALT, CPT, IPT, non-adherence to ART, month on ART, ART regimen, and NNRTI
43.	Chang et al., (2015) [116]	Retrospective cohort		2004–2012	8	32,611	22,106 (67.8)	2021 (6.2)	Sex, ART at enrolment year, ART regimen, WHO staging, CD4 count, viral load, anaemia, and ART adherence
44.	Bjerrum et al., (2016) [117]	Prospective cohort		2013–2014	7	473	304 (64.3)	60 (12.7)	Age, sex, CD4 count, cough ≥2 weeks, and fever ≥2 weeks
45.	Temesgen et al., (2019) [40]	Retrospective cohort		2012–2017	7	492	264 (53.7)	83 (16.9)	Sex, CD4 count, WHO staging, functional status, Hgb, OIs, CPT, and IPT
46.	Mupfumi et al., (2018) [118]	Retrospective cohort		2008–2011	7	254	172 (67.7)	13 (5)	Sex, age, Hgb, CD4 count, viral load, and hepatitis B infection
47.	Hanrahan et al., (2010) [29]	Prospective cohort		2003–2008	9	3, 456	2704 (78.2)	226 (6.5)	On HAART, CD4 count, CPT use, income, employment status, and smoking ever
48.	Melkamu et al., (2013) [119]	Case-Control Study		2011–2012	9	357	192 (53.8)	NR	Marital status, educational status, having diabetes mellitus, WHO clinical staging, and having separate kitchen
49.	Gedle et al., (2017) [120]	Cross-sectional		April–June 2016	10	323	204 (63.2)	142 (35.9)	Residence, educational status, income, presence of animals, presence of toilet, source of water, WHO staging, and CD4 count
50.	Kyeyune et al., (2014) [97]	Cross-sectional		2010–2012	9	400	277 (69.3)	260 (65)	Sex, CD4 count, age, HART status, educational status, and employment status
51.	Ageru et al., (2019) [121]	Cross-sectional		October–December 2016	10	411	258 (62.8)	150 (36.5)	Sex, marital status, educational level, HAART status, year live with virus, frequency of eating, CD4 count, and infection with intestinal parasite
52.	Chen et al., (2019) [122]	Prospective cohort		2007–2009	8	572	334 (58.4)	75 (13.1)	Loss of appetite, handgrip strength, sphygmomanometer test
53.	Hussen et al. (2017) [123]	Retrospective cohort		2006–2011	8	340	200 (58.8)	83 (24.4)	Educational level, baseline HAART, and INH prophylaxis

*ALT* Alanine Transaminase, *ART* Antiretroviral Therapy, *AST* Aspartate Aminotransferase, *CD4* Cluster of Differentiation 4, *CPT* Cotrimoxazole Preventive Therapy, *HAART* Highly Active Antiretroviral Therapy, *Hgb* Hemoglobin, *HIV* Human Immunodeficiency Virus, *INH* Isoniazid, *IPT* Isoniazid Preventive Therap, *MUAC* Mid Upper Arm Circumference, *NNRTI* Non-Nucleoside Reverse Transcriptase Inhibitor, *NR* Not reported, *OI* Opportunistic Infection, *RNA* Ribonucleic Acid, *TB* Tuberculosis, *TLC* Total Lymphocyte count, and *WHO* World Health Organization

### Quality appraisal results

NOS quality scores ranged from five to nine for cohort studies and eight to ten for cross-sectional studies (Table 1). The mean quality score of the included studies was 7.34 (SD: 0.14). More than two-thirds (71.7%) of the included studies had good quality. Fair or poor quality scores of the cohort studies were mainly due to the following reasons: lack of descriptions of loss to follow-up (*n* = 14, 29.8%), shorter follow-up period (*n* = 30, 63.8%), lack of description of the derivation of the exposed group (*n* = 19, 40.4%), and lack description of the derivation of the non-exposed group (*n* = 16, 34%). All included studies controlled confounders through multivariable regression analysis. However, most of the cohort studies employed a single-arm study design (no control group). From 47 cohort studies included in our review, 14 (29.8%) of the studies lacked a description of the loss to follow-up. Furthermore, six cohort studies reported that their loss to follow-up rate was more than 20%. About 63.8% of the included cohort studies had a follow-up period of less than five years for mortality and/or less than two years for morbidities.

### Narrative analysis

#### The effects of undernutrition on mortality

Five studies [95, 98, 102, 112, 124], which were not suitable for the meta-analysis, were included in the narrative analysis. A multi-county based retrospective cohort study involving 3749 adults living with HIV found that, as BMI increased in one unit, the hazard of death was reduced by 8% (AHR: 0.92, 95% CI: 0.87, 0.96) [98]. A Kenyan retrospective cohort study followed 71 adults living with HIV with a median follow up time of 201 days found that as BMI increased in one unit, the hazard of death decreased by 18% (AHR: 0.82, 95% CI: 0.68, 0.99) [95]. Another retrospective cohort study including 374 adults living with HIV from Uganda reported that adults living with HIV who had BMI < 17.5 kg/m^2^ were six-fold (AHR: 6.11, 95% CI: 2.3, 16.2) more likely to die as compared to well-nourished adults living with HIV. This study also documented that HIV positive adults, who had BMI between 17.5 and 18.49 kg/m^2^, were four times (AHR: 4.5, 95% CI: 1.54, 13.32) more likely to die than well-nourished adults living with [102]. Furthermore, a cross-sectional study involving 1507 participants conducted in Malawi reported that mild (AOR: AOR: 2.1, 95% CI: 1.2, 3.8), moderate (AOR: 2.4, 95% CI: 1.7, 6.3) and severe (AOR: 6, 95% CI: 4.6, 12.7) undernutrition were significantly associated with mortality [112]. Lastly, a cross-sectional study involving 947 adults living with HIV reported from South Africa found that severe undernourishment (AOR: 3.71, 95% CI: 1.89, 7.29) and BMI between 16 and 18.49 kg/m^2^ (AOR: 2.35, 95% CI: 1.3, 4.26) were significantly associated with mortality in PLHIV [124].

#### The effects of undernutrition on tuberculosis

From the 15 studies assessed respecting the effects of undernutrition on tuberculosis (TB), five studies were not suitable for meta-analysis [115–119]. A Tanzanian prospective cohort study followed 67,685 adults living with HIV with a median follow-up time of 24 months indicated that patients living with HIV with a BMI < 17 kg/m^2^ (AHR: 1.96, 95% CI: 1.83, 2.09), and BMI between 17 and 18.49 kg/m^2^ (AHR: 1.69, 95% CI: 1.58, 1.8) were at higher risk of TB [115]. An additional retrospective cohort study on 32,611 Nigerian adults living with HIV noted that within a median follow-up time of 29.2 months severely underweight [(BMI < 16 kg/m^2^ (AHR: 3.85, 95% CI: 2.75, 5.38)], and underweight [(BMI: 16–18.49 kg/m^2^ (AHR: 2.18, 95% CI: 1.80, 2.65)] adults living with HIV had a higher risk to be diagnosed with TB [116]. Furthermore, a retrospective cohort study conducted among 254 adults living with HIV in Botswana documented that, as BMI increased in one unit, the risk of TB reduced by 19% (AHR: 0.81, 95% CI 0.66, 1.00: *P* = 0.05) [118]. A case-control study undertaken with 357 adults living with HIV in Ethiopia reported that undernourished HIV positive adults had a higher risk of TB (AOR: 3.8, 95% CI: 2.39, 6.08) [119]. Another prospective cohort study done among 473 adults living with HIV in Ghana found that undernutrition significantly increased the risk of TB (AOR: 2.51, 95% CI: 1.15, 5.51) [117].

#### The effects of undernutrition on other morbidities

Five studies reported the effects of undernutrition on various morbidities in PLHIV [97, 120–123]. Two cross-sectional studies from Uganda involved 400 participants [97], and Ethiopia involved 411participants [121] showed that undernutrition significantly increased the risk of anaemia among PLHIV with prevalence ratio (PR): 2.43 (95% CI: 1.01, 5.26) and AOR: 2.96 (95% CI: 1.36, 6.39), respectively. A cross-sectional study conducted among 323 Ethiopian adults living with HIV reported that undernutrition was significantly associated with parasitic intestinal infections (AOR: 2.59, 95% CI: 1.36, 4.95) [120]. Furthermore, a Zambian prospective cohort study involving 572 participants found that moderate wasting was significantly associated with AIDS-defining illnesses (AOR: 2.40, 95% CI: 1.13, 5.10) [122]. At last, a retrospective cohort study conducted with 340 Ethiopian adults living with HIV showed that undernutrition was a significant risk of OIs (AHR: 2.27, 95% CI: 1.4, 3.6) [123].

### Meta-analysis of the effects of undernutrition on mortality

**Fig. 3:**
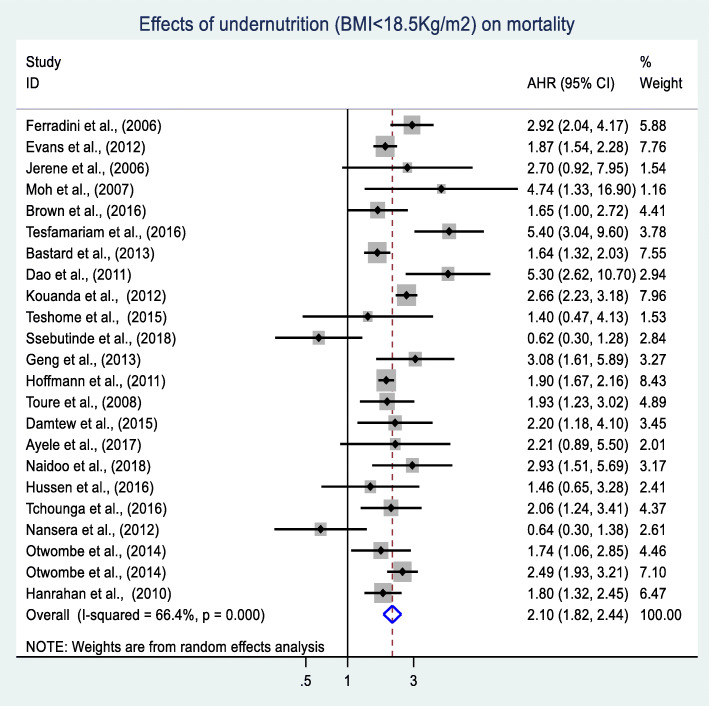
Forest plot of the effects of undernutrition on mortality among adults living with HIV in SSA

A total of 30 cohort studies were included in the meta-analysis. Of these, 23 studies reporting nutritional status as well-nourished (BMI between 18.5 kg/m^2^ and 24.9 kg/m^2^) and undernourished (BMI < 18.5 kg/m^2^) were analyzed together. The remaining seven studies reporting undernutrition based on the three categories of mild, moderate, and severe were addressed in the subgroup analyses (Table 2). Of the 23 studies included in the meta-analysis, 17 studies showed that undernutrition has a significant effect on mortality in adults living with HIV [18, 21, 24, 25, 27–31, 34, 101, 105, 106, 110, 111]. However, six studies reported that undernutrition has no significant effect on mortality in this population [19, 35, 37–39, 99]. Finally, the pooled AHR of 23 cohort studies involving 125,790 individuals showed that undernourished adults living with HIV were two-fold (AHR: 2.1, 95% CI: 1.8, 2.4) more likely to die as compared to their well-nourished counterparts. The included studies exhibited substantial heterogeneity (I^*2*^ = 66.4% and Cochrane chi-squared test *p*-value < 0.001). As a result, a random-effects meta-analysis model was conducted to estimate the final pooled effect size (Fig. 3).

**Table 2:** Subgroup analyses of the effect of undernutrition on mortality among adults living with HIV in SSA, between 2002 and 2019

Variables	Subgroup	No of studies	Population (N)	AHR (95%CI)	(I^**2**^ (%) and Cochrane chi-squared test ***p***-value)
Severity of undernutrition	Severe	6	66, 110	2.3 (1.9, 2.8)	(45.5, 0.102)
	Moderate	2	42, 308	1.8 (1.5, 2.5)	(52.3, 0.145)
	Mild	4	50, 754	1.4 (1.1, 1.8)	(60.9, 0.053)
Geographical locations	Eastern Africa	9	14, 601	1.8 (1.1, 3.0)	(75.9, < 0.001)
	Western Africa	3	16, 611	2.5 (1.9, 3.3)	(23, 0.273)
	Sothern Africa	8	36, 303	2.1 (1.8, 2.3)	(35.3, 0.147)
	Multicounty	3	58, 275	2.4 (1.3, 4.4)	(80, 0.007)
Sample size	≥ 5, 470	6	103, 441	1.8 (1.5, 2.3)	(79.3, < 0.001)
	< 5, 470	17	22, 349	2.3 (1.9, 2.9)	(56.6, 0.002)
Publication year	≤ 2012	9	45, 657	2.3 (1.9, 2.7)	(65.6, 0.003)
	> 2012	14	80, 133	1.9 (1.5, 2.4)	(68.2, < 0.001)
Quality score	Good	13	93, 236	2.05 (1.7, 2.5)	(73.3, < 0.001)
	Fair/ poor	10	32, 554	2.2 (1.7, 2.8)	(51.5, 0.029)

**Fig. 4:**
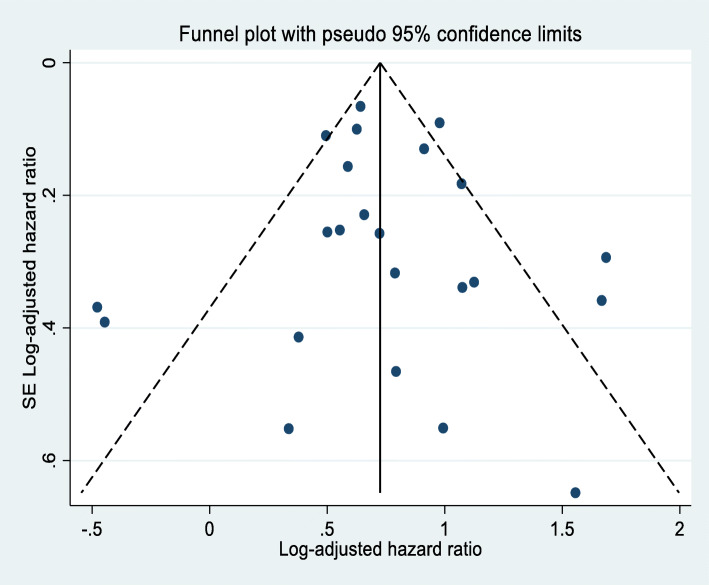
Funnel plot of the effects of undernutrition on mortality among adults living with HIV in SSA

The possible sources of heterogeneity were explored using a meta-regression model considering the following continuous variables as moderators: publication year, sample size, and quality. None of these factors were significantly associated with heterogeneity. Publication bias was assessed using a funnel plot. Since the funnel plot had a symmetric inverted shape, it is unlikely that there is publication bias (Fig. 4). To confirm this finding, objective statistical tests (Begg’s rank correlation and Egger’s linear regression tests) were conducted, which confirmed that there was no publication bias among studies used to estimate the effect of undernutrition on mortality with *p* = 0.5 and *p* = 0.8.

### Subgroup analyses of effects of undernutrition on mortality

The subgroup analyses of this review showed that severely undernourished adults living with HIV were at higher risk of death (AHR: 2.3, 95% CI: 1.9, 2.8) as compared to mildly undernourished adults living with HIV (using six studies) [20, 100, 104, 107–109]. However, the mortality rate between moderately undernourished (two studies) and mildly undernourished (four studies) adults living with HIV was not statistically significant [(AHR: 1.8, 95% CI: 1.5, 2.5) [20, 107], and (AHR: 1.4, 95% CI: 1.1, 1.8)] [20, 30, 103, 107], respectively. Moreover, undernutrition on mortality is exacerbated in Western Africa as compared to other parts of SSA (AHR: 2.5, 95% CI: 1.9, 3.3) [25, 27, 30]. The subgroup analyses also indicated that undernutrition has a more significant effect on mortality in studies published before 2012 (AHR: 2.3, 95% CI: 1.9, 2.7) [24, 25, 27–30, 99, 101, 106] (Table 2).

### Meta-analysis of effects of undernutrition on TB

**Fig. 5:**
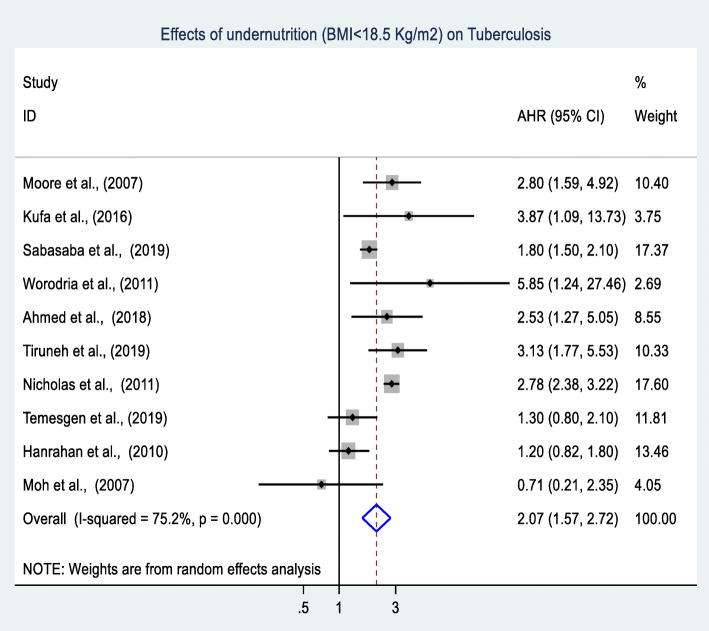
Forest plot of the effects of undernutrition on TB among adults living with HIV in SSA

**Fig. 6:**
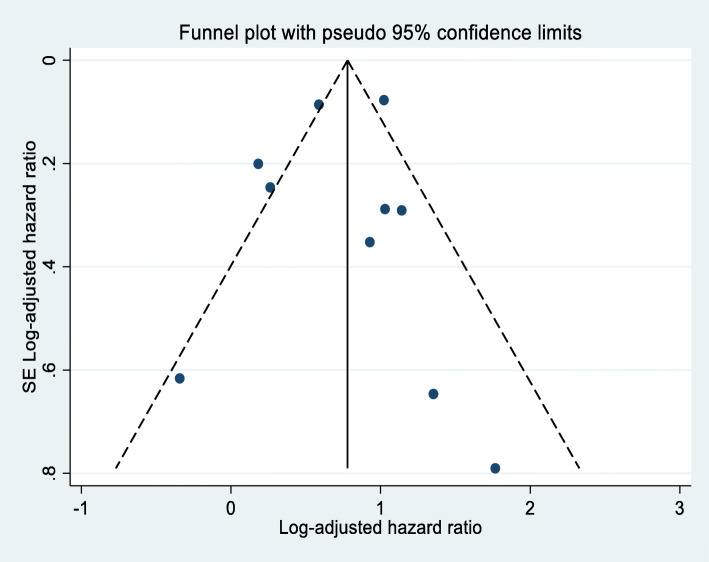
Funnel plot of the effects of undernutrition on TB among adults living with HIV in SSA

A total of ten cohort studies involving 104,387 adults living with HIV were included in the meta-analysis. Of these ten studies, seven studies found that undernutrition has a significant effect on the occurrence of TB [26, 32, 33, 36, 96, 113, 114]. The remaining three studies found that undernutrition has no significant effect on the occurrence of TB [25, 28, 40]. The final overall pooled effect found that undernutrition has a significant effect on the occurrence of TB among adults living with HIV (AHR: 2.1, 95% CI: 1.6, 2.7) (Fig. 5). Significant heterogeneity (I^2^ = 75.2% and Cochrane chi-squared test *p*-value < 0.001) was observed; therefore, a random-effects meta-analysis model was computed to estimate the pooled effect. To investigate the possible sources of heterogeneity, a univariate random-effects meta-regression was done using continuous variables of publication year, sample size, quality as covariates [(Coefficient: − 0.03, p: 0.524), (Coefficient: − 2.71, p: 0.736) and (Coefficient: 0.31: 0.307) respectively]. Finally, publication bias between the included studies was also assessed using the funnel plot (Fig. 6) with confirmatory tests of publication bias done using Begg’s rank correlation and Egger’s linear regression tests. Accordingly, both test sets indicated there was no publication bias across included studies (*p* = 0.325 and *p* = 0.767).

### Subgroup analyses of effects of undernutrition on TB

Subgroup analyses were performed based on geographical locations, sample size, and year of publication. Our subgroup analyses indicated that undernutrition has a higher effect (AHR: 2.2, 95% CI: 1.6: 2.9) on the occurrence of TB among adults living with HIV in studies done in Eastern Africa as compared to other parts of SSA. Interestingly, insignificant heterogeneity (I^2^ = 51%, *p*-value = 0.069) was observed between studies conducted in Eastern Africa. The subgroup analysis based on publication year and sample size found no significant difference in undernutrition’s effect on the occurrence of TB among adults living with HIV. However, a considerable heterogeneity difference was observed across these factors (Table 3).

**Table 3:** Subgroup analyses of the effect of undernutrition on TB among adults living with HIV in SSA, between 2002 and 2019

Variables	Subgroup	No of studies	Population (N)	AHR (95%CI)	(I^**2**^ (%) and Cochrane chi-squared test ***p***-value)
Geographical locations	Eastern Africa	6	71, 182	2.2 (1.6, 2.9)	51, 0.069
	Other countries	4	33, 205	1.8 (0.9, 3.5)	84.9, < 0.001
Sample size	≥ 1, 000	4	101, 199	2.02 (1.4, 2.9)	87.8, < 0.001
	< 1, 000	6	3, 188	2.2 (1.3, 3.6)	56.8, 0.041
Publication year	≤ 2012	5	33, 832	2.02 (1.2, 3.4)	80.7, < 0.001
	> 2012	5	70, 555	2.02 (1.5, 2.8)	46.8, 0.111

## Discussion

Despite encouraging recent scale-up of ART, nutrition-related early mortality from HIV is a persistent concern in SSA. Therefore, this systematic review and meta-analysis aimed to estimate the pooled effects of undernutrition on mortality and morbidity among adults living with HIV in SSA. To the best of our knowledge, this review is the first of its kind. The findings of this review highlighted that undernutrition significantly increases the risk of mortality and morbidity in adults living with HIV in SSA, and that, as the degree of undernutrition became more severe, mortality rate also increased.

The overall pooled estimate of 23 cohort studies involving 125,790 adults living with HIV indicated that undernourished adults living with HIV were two times (AHR: 2.1, 95% CI: 1.8, 2.4) more likely to die as compared to their well-nourished counterparts. Different mechanisms could explain the observed association between undernutrition and mortality. Undernutrition significantly impairs the immune response, which could increase the risk of developing and recurrence of OIs in the early phase of ART and ultimately contributing to early mortality. There is evidence that malnutrition adversely affects both innate and adaptive immunity systems, which are essential for defense against infections [125]. OIs are the leading cause of mortality among PLHIV, being responsible for more than 94% of AIDS-related deaths [126, 127].

Another mechanism explaining the effect of undernutrition on mortality could be due to its impact on the adherence level of ART. Different studies revealed that undernutrition is significantly associated with poor ART adherence levels [128, 129]. The effectiveness of HIV treatment depends on ART drug adherence. PLHIV are recommended to take their medications continuously and daily [130] as ART drug adherence is the proximal predictor of mortality [131, 132]. An additional possible explanation for the observed effect of undernutrition on mortality might be due to its impact on ART treatment failure [133]. PLHIV, who had a history of treatment failure or not taking their ART drug properly, are at higher risk of death as compared to those who had good treatment response or good adherence level to their ART drugs [134, 135].

This review found that, as the degree of undernutrition became more severe, mortality rate also increased. This dose-response relationship of undernutrition and mortality could result from the severity of malnourishment increasing the occurrence of OIs, which are the leading cause of mortality among PLHIV [16]. It is postulated that malnutrition and infection are interrelated in a vicious cycle [136]. Infections contribute to malnutrition through different means: increased metabolic demand, loss of appetite, and decreased absorption. On the contrary, malnutrition increases the risk of infections by causing immune deficiency, resulting in the persistence of malnutrition as the most common cause of immunodeficiency [16, 137].

The second outcome of this review demonstrated that undernutrition significantly increased the risk of developing morbidities among adults living with HIV. A meta-analysis of ten cohort studies involving 104,387 adults living with HIV showed that undernourished adults living with HIV were twice as likely to develop TB as compared to their well-nourished counterparts. Our finding is in line with a systematic review of cohort studies, which reported that a higher risk of TB was observed among adults with BMI < 18.5 kg/m^2^ [138]. This finding might be due to malnutrition’s weakening of the immune system and the concomitant increased risk of comorbidities including TB infections [139].

The relationship between malnutrition and TB has been well documented [140]. The bidirectional relationship is more accentuated among adults living with HIV, because HIV further weakened the immune system and increased the risk of TB. Evidence suggests that malnutrition increases the risk of disease progression from latent TB to active TB by weakening the immune system among adults living with HIV [141]. Besides, food insecurity may delay the health-seeking behavior, which results in late diagnosis and poor treatment adherence of TB [142]. This problem is more severe in SSA, where 68% of the PLHIV in 2018 lived [2, 4], and 23.2% of the world’s food-insecure people in 2015 lived [143]. On the other hand, TB can cause loss of appetite, malabsorption, and increase metabolic demand [144].

Finally, subgroup analyses confirmed that undernutrition appears to have a more deleterious effect on the occurrence of TB among adults living with HIV in Eastern Africa as compared to other parts of SSA. The possible explanation for this variance might be due to the economic differences across included countries. Likewise, the studies included in Eastern Africa were obtained from Ethiopia, Tanzania, and Uganda. According to the 2019 World Bank report, all three countries were classified as low-income [145]. Even though the clear association is not well known, poverty is widely recognized as the leading risk factor for TB [129]. Moreover, the accentuated effect of undernutrition on TB could be due to the clinical profile of the participants included in primary studies. As an example, more than half (54.6%) of the participants involved in an Ethiopian study were classified as WHO clinical stage III and IV [33]. Furthermore, about 44.5% of the participants involved in a Tanzanian study were classified as WHO clinical stage III and IV [96]. The more advanced HIV/AIDS disease stage coincides with the increased occurrence and the recurrence of OIs, including TB [146]. According to the recent Ethiopian National ARV treatment guidelines, adults living with HIV presenting with pulmonary TB are classified as WHO stage III and with extra pulmonary TB are classified as WHO stage IV [147].

### What does this study add to what is known?

Although different clinical trials showed nutritional interventions have no effect on mortality [16, 148, 149], the lancet HIV commentary paper strongly recommended that nutritional supplementations for patients on ART should be continued because it could increase body weight, hasten physical and functional recovery, and improve work capacity and quality of life [150]. Similarly, the WHO recommends that severely undernourished adults living with HIV should be treated with therapeutic foods. Moderately undernourished adults living with HIV can be treated with supplementary foods. Besides, nutritional assessments for PLHIV should be done regularly [151]. Although undernutrition is the proximal risk factor increasing mortality and morbidity among adults living with HIV [152], a comprehensive review estimating the effects of undernutrition on mortality and morbidity in this vulnerable population in SSA is lacking. Our results showed that undernutrition increased the risk of death and TB among adults living with HIV by two-fold. For policy makers and program planners, highly credible evidence obtained from systematic reviews and meta-analyses are vital. Therefore, findings from this review may be used to update the nutritional guidelines used for the management of PLHIV by different stakeholders, especially in limited-resource settings.

### Strengths and limitations

There are a number of strengths with this review. An extensive search strategy was undertaken. Explicit inclusion and exclusion criteria regarding population, exposure, control, and outcomes were used. Three authors were involved in the quality assessment. A homogenous exposure category (BMI < 18.5 kg/m^2^) was used rather than including studies that used different categories for the meta-analysis. Attempts were made to control the confounders by taking the AHR for the meta-analysis. Since the included studies exhibited considerable heterogeneity, advanced statistical analyses such as meta-regression were performed to identify possible heterogeneity sources. Most of the included studies used measured weight and height to calculate BMI from medical records rather than self-reported weight and height thereby avoiding recall biases.

Despite the above mentioned strengths, this review has some constraints that must be considered before interpreting results. This review included some studies with small sample sizes, potentially influencing findings. Our search limited to studies published in the English language, which may have resulted in the exclusion of a few essential studies. Many studies reported BMI in different categories making it difficult to include all studies in our meta-analysis. However, this variance has been addressed through subgroup analyses and qualitative analysis. Despite the use of AHR for our meta-analysis, most of the included primary studies used retrospective data. Thus, these studies did not include some important nutritional variables like socioeconomic status and dietary diversity. Furthermore, the actual effect of undernutrition on mortality could be confounded by undiagnosed acute diseases. This review included studies reported from 13 SSA countries and six multicounty based studies, which may yield underrepresentation of other SSA countries. Lastly, the majority of included studies used baseline BMI, but it changed continuously over time. Therefore, this result may not reflect the actual effects of malnutrition on mortality and morbidity.

## Conclusion

This review found that undernutrition has significant effects on mortality and morbidity among adults living with HIV. As the degree of undernutrition became more severe, mortality rate also increased. Based on our findings, we recommended that nutritional assessment among adults living with HIV needs to be done regularly. Moreover, early screening of morbidities like TB among undernourished adults living with HIV is recommended. Furthermore, besides the management of malnutrition, comprehensive nutritional counselling to improve diet by consuming locally available needs to be reinforced at each visit for HIV care. Further studies are needed to examine the impact of nutritional interventions to improve nutritional status on mortality and morbidities among adults living with HIV. Finally, further follow-up studies considering malnutrition as exposure variable are needed to examine its actual effects on mortality and morbidities.

## Supplementary Information

**Additional file 1.** PRISMA 2009 Checklist.

**Additional file 2.** 1. PubMed search history. 2. EMBASE search history (Elsevier). 3. Scopus search history. 4. Search from CINHAL.

## Data Availability

The data sets used and/or analyzed for this review are available from the corresponding author on reasonable request.

## Abbreviations

AHR: Adjusted hazard ratio
AIDS: Acquired Immunodeficiency syndrome
ART: Antiretroviral therapy
BMI: Body mass index
CI: Confidence interval
HAART: Highly active antiretroviral treatment
HIV: Human immunodeficiency virus
LMICs: Low and middle-income countries
NOS: Newcastle-Ottawa scale
OIs: Opportunistic infections
PLHIV: People living with human immunodeficiency virus
SSA: Sub-Saharan Africa
TB: Tuberculosis
WHO: World health Organization
