# Supplementary material for: Effects of undernutrition on mortality and morbidity among adults living with HIV in sub-Saharan Africa: a systematic review and meta-analysis
Source: BMC Infect Dis. 2021 Jan 4;21:1. doi: 10.1186/s12879-020-05706-z (PMC7780691; doi:10.1186/s12879-020-05706-z)
Supplement: Supplementary file 2 — Additional file 2. 1. PubMed search history. 2. EMBASE search history (Elsevier). 3. Scopus search history. 4. Search from CINHAL. [file 12879_2020_5706_MOESM2_ESM.docx]

1. **PubMed search history**

| **Search** | **Query** | **Items found** |
| --- | --- | --- |
| #8 | #4 AND #3 AND #2 AND #1 Filters: Publication date from 2002/01/01 to 2019/11/19; Humans; English | [970](https://www-ncbi-nlm-nih-gov.ezproxy.lib.uts.edu.au/pubmed/?cmd=HistorySearch&querykey=10) |
| #7 | #4 AND #3 AND #2 AND #1 Filters: Humans; English | [1097](https://www-ncbi-nlm-nih-gov.ezproxy.lib.uts.edu.au/pubmed/?cmd=HistorySearch&querykey=9) |
| #6 | #4 AND #3 AND #2 AND #1 Filters: Humans | [1137](https://www-ncbi-nlm-nih-gov.ezproxy.lib.uts.edu.au/pubmed/?cmd=HistorySearch&querykey=8) |
| #5 | #4 AND #3 AND #2 AND #1 | [1297](https://www-ncbi-nlm-nih-gov.ezproxy.lib.uts.edu.au/pubmed/?cmd=HistorySearch&querykey=7) |
| #4 | Angola OR Benin OR Botswana OR Burkina Faso OR Burundi OR Cameroon OR Cape Verde OR Central African Republic OR Chad OR Comoros OR Republic of the Congo OR Democratic Republic of the Congo OR Cote d'Ivoire OR Djibouti OR Equatorial Guinea OR Eritrea OR Ethiopia OR Gabon OR The Gambia OR Ghana OR Guinea OR Guinea-Bissau OR Kenya OR Liberia OR Madagascar OR Malawi OR Mali OR Mauritania OR Mauritius OR Mozambique OR Namibia OR Niger OR Nigeria OR Rwanda OR Sao Tome and Principe OR Senegal OR Seychelles OR Sierra Leone OR Somalia OR South Africa OR South Sudan OR Sudan OR Swaziland OR Tanzania OR Togo OR Uganda OR Zambia OR Zimbabwe | [529753](https://www-ncbi-nlm-nih-gov.ezproxy.lib.uts.edu.au/pubmed/?cmd=HistorySearch&querykey=4) |
| **#3** | HIV Infections[MH] OR hiv[tw] OR hiv-1[tw] OR hiv-2[tw] OR hiv-infect*[tw] OR human immunodeficiency virus[tw] OR human immunedeficiency virus[tw] OR human immuno-deficiency virus [tw] OR human immune-deficiency virus[tw] OR acquired immunodeficiency syndrome [tw] OR acquired immunedeficiency syndrome[tw] OR acquired immunodeficiency syndrome[tw] OR acquired immune-deficiency syndrome[tw] OR “Sexually Transmitted Diseases, Viral”[MeSH:NoExp] | [402058](https://www-ncbi-nlm-nih-gov.ezproxy.lib.uts.edu.au/pubmed/?cmd=HistorySearch&querykey=3) |
| **#2** | Mortality[MH] OR Mortalit*[TIAB] OR incidence [TIAB] OR survival [TIAB] OR death rate[TIAB] OR risk factors[TIAB] OR time to death[TIAB] OR Case fatality rate[TIAB] OR determinates[TIAB] OR Mortality rate[TIAB] OR predictors[TIAB] OR [Opportunistic Infections](https://www.ncbi.nlm.nih.gov/mesh/68009894) [MH] OR AIDS Related opportunistic Infections[MH] OR morbidit*[TIAB] OR opportunistic infect*[TIAB] OR hospital admissions[TIAB] OR hospitalization[TIAB] OR herpes zoster[TIAB] OR bacterial pneumonia[TIAB] OR pulmonary TB[TIAB] OR extra-pulmonary TB[TIAB] OR tuberculosis[TIAB] OR TB[TIAB] OR oral candidiasis[TIAB] OR oesophageal candidiasis[TIAB] OR mouth ulcer[TIAB] OR diarrh*[TIAB] OR pneumocystis pneumonia[TIAB] OR central nervous system toxoplasmosis[TIAB] OR toxoplasmosis[TIAB] OR cryptococcal meningitis[TIAB] OR non-Hodgkins lymphoma[TIAB] OR Kaposi’s sarcoma[TIAB] OR cervical cancer[TIAB] OR herpes simplex[TIAB] OR cytomegalovirus[TIAB] OR AIDS defining disease[TIAB] | [3250509](https://www-ncbi-nlm-nih-gov.ezproxy.lib.uts.edu.au/pubmed/?cmd=HistorySearch&querykey=2) |
| **#1** | Malnutrition[MH] OR Body Mass Index[tw] OR Malnourishm*[tw] OR undernutrition[tw] OR Nutritional deficienc*[tw] OR Nutritional status[tw] OR BMI[tw] OR underweight[tw] OR stunting[tw] OR wasting[tw] OR micronutrient deficienc*[tw] | \| [451855](https://www-ncbi-nlm-nih-gov.ezproxy.lib.uts.edu.au/pubmed/?cmd=HistorySearch&querykey=1) \|  \| \| --- \| --- \| |

1. **EMBASE search history (Elsevier)**

| **No** | **Query** | **Results** |
| --- | --- | --- |
| #8 | #7 AND 'human'/de | [1,302](https://www-embase-com.ezproxy.lib.uts.edu.au/) |
| #7 | #5 AND (2002:py OR 2003:py OR 2004:py OR 2005:py OR 2006:py OR 2007:py OR 2008:py OR 2009:py OR 2010:py OR 2011:py OR 2012:py OR 2013:py OR 2014:py OR 2015:py OR 2016:py OR 2017:py OR 2018:py OR 2019:py) AND [english]/lim | [1,337](https://www-embase-com.ezproxy.lib.uts.edu.au/) |
| #6 | #5 AND (2002:py OR 2003:py OR 2004:py OR 2005:py OR 2006:py OR 2007:py OR 2008:py OR 2009:py OR 2010:py OR 2011:py OR 2012:py OR 2013:py OR 2014:py OR 2015:py OR 2016:py OR 2017:py OR 2018:py OR 2019:py) | [1,365](https://www-embase-com.ezproxy.lib.uts.edu.au/) |
| #5 | #1 AND #2 AND #3 AND #4 | [1,500](https://www-embase-com.ezproxy.lib.uts.edu.au/) |
| #4 | 'angola' OR 'benin' OR 'botswana' OR 'burkina faso' OR 'burundi' OR 'cameroon' OR 'cape verde' OR 'central african republic' OR 'chad' OR 'comoros' OR 'republic of the congo' OR 'democratic republic of the congo' OR 'cote d ivoire' OR 'djibouti' OR 'equatorial guinea' OR 'eritrea' OR 'ethiopia' OR 'gabon' OR 'the gambia' OR 'ghana' OR 'guinea' OR 'guinea-bissau' OR 'kenya' OR 'liberia' OR 'madagascar' OR 'malawi' OR 'mali' OR 'mauritania' OR 'mauritius' OR 'mozambique' OR 'namibia' OR 'niger' OR 'nigeria' OR 'rwanda' OR 'sao tome and principe' OR 'senegal' OR 'seychelles' OR 'sierra leone' OR 'somalia' OR 'south africa' OR 'south sudan' OR 'sudan' OR 'swaziland' OR 'tanzania' OR 'togo' OR 'uganda' OR 'zambia' OR 'zimbabwe' | [713,318](https://www-embase-com.ezproxy.lib.uts.edu.au/) |
| #3 | 'human immunodeficiency virus infection'/exp OR 'human immunodeficiency virus infection' OR 'human immunodeficiency virus'/exp OR 'human immunodeficiency virus' OR hiv:ti,ab OR 'hiv-1':ti,ab OR 'hiv-2':ti,ab OR 'human immunodeficiency virus':ti,ab OR 'human immuno-deficiency virus':ti,ab OR 'human immunedeficiency virus':ti,ab OR 'human immune-deficiency virus':ti,ab OR 'acquired immune-deficiency syndrome':ti,ab OR 'acquired immunedeficiency syndrome':ti,ab OR 'acquired immunodeficiency syndrome':ti,ab OR 'acquired immuno-deficiency syndrome':ti,ab | [533,865](https://www-embase-com.ezproxy.lib.uts.edu.au/) |
| #2 | ('mortality'/exp OR 'mortality':ti,ab OR 'survival':ti,ab OR 'time to death' OR 'risk factors of mortality' OR 'predictors of mortality' OR 'mortality rate':ti,ab OR 'mortality rates':ti,ab OR 'death rate' OR 'case fatality rate' OR 'mortality determinates' OR 'mortality predictors' OR 'morbidity' OR 'morbidities') AND 'opportunistic infection'/exp OR 'opportunistic infection' OR 'opportunistic infections' OR 'aids related opportunistic infection' OR 'hospital admissions' OR 'hospitalization' OR 'herpes zoster' OR 'bacterial pneumonia' OR 'pulmonary tb' OR 'extra-pulmonary tb' OR 'tuberculosis' OR 'tb' OR 'oral candidiasis' OR 'oesophageal candidiasis' OR 'mouth ulcer' OR 'diarrhoea' OR 'diarrhea' OR 'pneumocystis pneumonia' OR 'central nervous system toxoplasmosis' OR 'cryptococcal meningitis' OR 'non-hodgkins lymphoma' OR 'kaposis sarcoma' OR 'cervical cancer' OR 'herpes simplex' OR 'cytomegalovirus' OR 'aids defining disease' | [1,333,445](https://www-embase-com.ezproxy.lib.uts.edu.au/) |
| #1 | 'malnutrition'/exp OR 'malnutrition':ti,ab OR 'body mass':ti,ab OR 'body mass index':ti,ab OR 'nutritional deficiency':ti,ab OR 'bmi':ti,ab OR 'underweight'/exp OR 'underweight':ti,ab OR 'thinness':ti,ab OR 'stunting':ti,ab OR 'wasting' OR 'wasting syndrome':ti,ab OR 'micronutrient deficiency':ti,ab OR 'nutritional status':ti,ab OR 'deficient nutrition':ti,ab OR 'malnourishment':ti,ab OR 'severe acute malnutrition':ti,ab OR 'underfeeding':ti,ab OR 'undernourishment':ti,ab OR 'undernutrition':ti,ab | [679,986](https://www-embase-com.ezproxy.lib.uts.edu.au/) |

1. **Scopus search history**

| **No** | **Query** | **Results** |
| --- | --- | --- |
| **#7** | ("malnutrition" OR "undernutrition" OR "malnourishmt*" OR "nutritional deficienc*" OR "nutritional status" OR "Body Mass Index" OR "BMI" OR "micronutrient deficienc*" OR "stunting" "wasting" OR "underweight") AND ("Mortalit*" OR "opportunistic infection*" OR "AIDS Related opportunistic infection*" OR "survival status" OR "death rate" OR "case fatality rate" OR "mortality determina*" OR "mortality rat*" OR "mortality predict*" OR "risk of mortality" OR "morbidit*" OR "hospital admissions" OR "hospitalization" OR "herpes zoster" OR "bacterial pneumonia" OR "pulmonary TB" OR "extra-pulmonary TB" OR "oral candidiasis" OR "oesophageal candidiasis" OR "mouth ulcer" OR "diarrhoea" OR "pneumocystis pneumonia" OR "central nervous system toxoplasmosis" OR "cryptococcal meningitis" OR "non-Hodgkins lymphoma" OR "Kaposi's sarcoma" OR "cervical cancer" OR "herpes simplex" OR "cytomegalovirus") AND ("HIV infect*" OR "hiv" OR "human immunodeficiency virus" OR "human immunedeficiency virus" OR "human immuno-deficiency virus" OR "human immune-deficiency virus" OR "acquired immunodeficiency syndrome" OR "acquired immunedeficiency syndrome" OR "acquired immunodeficiency syndrome" OR "acquired immune-deficiency syndrome" OR "hiv-positive" OR "sexually transmitted diseases") AND ("Angola" OR "Benin" OR "Botswana" OR "Burkina Faso" OR "Burundi" OR "Cameroon" OR "Cape Verde" OR "Central African Republic" OR "Chad" OR "Comoros" OR "Republic of the Congo" OR "Democratic Republic of the Congo" OR "Cote d'Ivoire" OR Djibouti OR "Equatorial Guinea" OR "Eritrea" OR "Ethiopia" OR "Gabon" OR "The Gambia" OR "Ghana" OR "Guinea" OR "Guinea-Bissau" OR "Kenya" OR "Liberia" OR "Madagascar" OR "Malawi" OR "Mali" OR "Mauritania" OR "Mauritius" OR "Mozambique" OR "Namibia" OR "Niger" OR "Nigeria" OR "Rwanda" OR "Sao Tome and Principe" OR "Senegal" OR "Seychelles" OR "Sierra Leone" OR "Somalia" OR "South Africa" OR "South Sudan" OR "Sudan" OR "Swaziland" OR "Tanzania" OR "Togo" OR "Uganda" OR "Zambia" OR "Zimbabwe") AND ( LIMIT-TO ( PUBYEAR,2019) OR LIMIT-TO ( PUBYEAR,2018) OR LIMIT-TO ( PUBYEAR,2017) OR LIMIT-TO ( PUBYEAR,2016) OR LIMIT-TO ( PUBYEAR,2015) OR LIMIT-TO ( PUBYEAR,2014) OR LIMIT-TO ( PUBYEAR,2013) OR LIMIT-TO ( PUBYEAR,2012) OR LIMIT-TO ( PUBYEAR,2011) OR LIMIT-TO ( PUBYEAR,2010) OR LIMIT-TO ( PUBYEAR,2009) OR LIMIT-TO ( PUBYEAR,2008) OR LIMIT-TO ( PUBYEAR,2007) OR LIMIT-TO ( PUBYEAR,2006) OR LIMIT-TO ( PUBYEAR,2005) OR LIMIT-TO ( PUBYEAR,2004) OR LIMIT-TO ( PUBYEAR,2003) OR LIMIT-TO ( PUBYEAR,2002) ) AND ( LIMIT-TO ( LANGUAGE,"English" ) ) | [1,917 document results](https://www-scopus-com.ezproxy.lib.uts.edu.au/search/history/results.uri?origin=searchhistory&shid=14) |
| **#6** | ("malnutrition" OR "undernutrition" OR "malnourishmt*" OR "nutritional deficienc*" OR "nutritional status" OR "Body Mass Index" OR "BMI" OR "micronutrient deficienc*" OR "stunting" "wasting" OR "underweight") AND ("Mortalit*" OR "opportunistic infection*" OR "AIDS Related opportunistic infection*" OR "survival status" OR "death rate" OR "case fatality rate" OR "mortality determina*" OR "mortality rat*" OR "mortality predict*" OR "risk of mortality" OR "morbidit*" OR "hospital admissions" OR "hospitalization" OR "herpes zoster" OR "bacterial pneumonia" OR "pulmonary TB" OR "extra-pulmonary TB" OR "oral candidiasis" OR "oesophageal candidiasis" OR "mouth ulcer" OR "diarrhoea" OR "pneumocystis pneumonia" OR "central nervous system toxoplasmosis" OR "cryptococcal meningitis" OR "non-Hodgkins lymphoma" OR "Kaposi's sarcoma" OR "cervical cancer" OR "herpes simplex" OR "cytomegalovirus") AND ("HIV infect*" OR "hiv" OR "human immunodeficiency virus" OR "human immunedeficiency virus" OR "human immuno-deficiency virus" OR "human immune-deficiency virus" OR "acquired immunodeficiency syndrome" OR "acquired immunedeficiency syndrome" OR "acquired immunodeficiency syndrome" OR "acquired immune-deficiency syndrome" OR "hiv-positive" OR "sexually transmitted diseases") AND ("Angola" OR "Benin" OR "Botswana" OR "Burkina Faso" OR "Burundi" OR "Cameroon" OR "Cape Verde" OR "Central African Republic" OR "Chad" OR "Comoros" OR "Republic of the Congo" OR "Democratic Republic of the Congo" OR "Cote d'Ivoire" OR Djibouti OR "Equatorial Guinea" OR "Eritrea" OR "Ethiopia" OR "Gabon" OR "The Gambia" OR "Ghana" OR "Guinea" OR "Guinea-Bissau" OR "Kenya" OR "Liberia" OR "Madagascar" OR "Malawi" OR "Mali" OR "Mauritania" OR "Mauritius" OR "Mozambique" OR "Namibia" OR "Niger" OR "Nigeria" OR "Rwanda" OR "Sao Tome and Principe" OR "Senegal" OR "Seychelles" OR "Sierra Leone" OR "Somalia" OR "South Africa" OR "South Sudan" OR "Sudan" OR "Swaziland" OR "Tanzania" OR "Togo" OR "Uganda" OR "Zambia" OR "Zimbabwe") AND ( LIMIT-TO ( PUBYEAR,2019) OR LIMIT-TO ( PUBYEAR,2018) OR LIMIT-TO ( PUBYEAR,2017) OR LIMIT-TO ( PUBYEAR,2016) OR LIMIT-TO ( PUBYEAR,2015) OR LIMIT-TO ( PUBYEAR,2014) OR LIMIT-TO ( PUBYEAR,2013) OR LIMIT-TO ( PUBYEAR,2012) OR LIMIT-TO ( PUBYEAR,2011) OR LIMIT-TO ( PUBYEAR,2010) OR LIMIT-TO ( PUBYEAR,2009) OR LIMIT-TO ( PUBYEAR,2008) OR LIMIT-TO ( PUBYEAR,2007) OR LIMIT-TO ( PUBYEAR,2006) OR LIMIT-TO ( PUBYEAR,2005) OR LIMIT-TO ( PUBYEAR,2004) OR LIMIT-TO ( PUBYEAR,2003) OR LIMIT-TO ( PUBYEAR,2002) ) | [1,931 document results](https://www-scopus-com.ezproxy.lib.uts.edu.au/search/history/results.uri?origin=searchhistory&shid=12) |
| **#5** | **#1 AND #2 AND #3 AND #4** | [2,112 document results](https://www-scopus-com.ezproxy.lib.uts.edu.au/search/history/results.uri?origin=searchhistory&shid=5) |
| **#4** | “Angola” OR “Benin” OR “Botswana” OR “Burkina Faso” OR “Burundi” OR “Cameroon” OR “Cape Verde” OR “Central African Republic” OR “Chad” OR “Comoros” OR “Republic of the Congo” OR “Democratic Republic of the Congo” OR “Cote d'Ivoire” OR Djibouti OR “Equatorial Guinea” OR “Eritrea” OR “Ethiopia” OR “Gabon” OR “The Gambia” OR “Ghana” OR “Guinea” OR “Guinea-Bissau” OR “Kenya” OR “Liberia” OR “Madagascar” OR “Malawi” OR “Mali” OR “Mauritania” OR “Mauritius” OR “Mozambique” OR “Namibia” OR “Niger” OR “Nigeria” OR “Rwanda” OR “Sao Tome and Principe” OR “Senegal” OR “Seychelles” OR “Sierra Leone” OR “Somalia” OR “South Africa” OR “South Sudan” OR “Sudan” OR “Swaziland” OR “Tanzania” OR “Togo” OR “Uganda” OR “Zambia” OR “Zimbabwe” | [3,020,727 document results](https://www-scopus-com.ezproxy.lib.uts.edu.au/search/history/results.uri?origin=searchhistory&shid=4) |
| **#3** | "HIV infect*"  OR  "hiv"  OR  "human immunodeficiency virus"  OR  "human immunedeficiency virus"  OR  "human immuno-deficiency virus"  OR  "human immune-deficiency virus"  OR  "acquired immunodeficiency syndrome"  OR  "acquired immunedeficiency syndrome"  OR  "acquired immunodeficiency syndrome"  OR  "acquired immune-deficiency syndrome"  OR  "hiv-positive"  OR  "sexually transmitted diseases" | [1,289,262 document results](https://www-scopus-com.ezproxy.lib.uts.edu.au/search/history/results.uri?origin=searchhistory&shid=3) |
| #2 | "Mortalit*" OR "opportunistic infection*" OR "AIDS Related opportunistic infection*" OR "survival status" OR "death rate" OR "case fatality rate" OR "mortality determina*" OR "mortality rat*" OR "mortality predict*" OR "risk of mortality" OR "morbidit*" OR "hospital admissions" OR "hospitalization" OR "herpes zoster" OR "bacterial pneumonia" OR "pulmonary TB" OR "extra-pulmonary TB" OR "oral candidiasis" OR "oesophageal candidiasis" OR "mouth ulcer" OR "diarrhoea" OR "pneumocystis pneumonia" OR "central nervous system toxoplasmosis" OR "cryptococcal meningitis" OR "non-Hodgkins lymphoma" OR "Kaposi's sarcoma" OR "cervical cancer" OR "herpes simplex" OR "cytomegalovirus" | [4,791,117 document results](https://www-scopus-com.ezproxy.lib.uts.edu.au/search/history/results.uri?origin=searchhistory&shid=2) |
| #1 | "malnutrition"  OR  "undernutrition"  OR  "malnourishmt*"  OR  "nutritional deficienc*"  OR  "nutritional status"  OR  "Body Mass Index"  OR  "BMI"  OR  "micronutrient deficienc*"  OR  "stunting"  "wasting"  OR  "underweight" | [40,633 document results](https://www-scopus-com.ezproxy.lib.uts.edu.au/search/history/results.uri?origin=searchhistory&shid=1) |

1. **Search from CINHAL**

| [**Search ID#**](javascript:__doPostBack('ctl00$ctl00$MainContentArea$MainContentArea$historyControl$ReorderHistoryLink','')) | **Search Terms** | **Results** |
| --- | --- | --- |
| S6 | Limiters - Published Date: 20020101-20191231 | 120 |
| S5 | S1 AND S2 AND S3 AND S4 | 124 |
| S4 | “Angola” OR “Benin” OR “Botswana” OR “Burkina Faso” OR “Burundi” OR “Cameroon” OR “Cape Verde” OR “Central African Republic” OR “Chad” OR “Comoros” OR “Republic of the Congo” OR “Democratic Republic of the Congo” OR “Cote d'Ivoire” OR Djibouti OR “Equatorial Guinea” OR “Eritrea” OR “Ethiopia” OR “Gabon” OR “The Gambia” OR “Ghana” OR “Guinea” OR “Guinea-Bissau” OR “Kenya” OR “Liberia” OR “Madagascar” OR “Malawi” OR “Mali” OR “Mauritania” OR “Mauritius” OR “Mozambique” OR “Namibia” OR “Niger” OR “Nigeria” OR “Rwanda” OR “Sao Tome and Principe” OR “Senegal” OR “Seychelles” OR “Sierra Leone” OR “Somalia” OR “South Africa” OR “South Sudan” OR “Sudan” OR “Swaziland” OR “Tanzania” OR “Togo” OR “Uganda” OR “Zambia” OR “Zimbabwe” | 69,581 |
| S3 | (MH "Human Immunodeficiency Virus") OR “HIV infect*” OR “hiv” OR “human immunedeficiency virus” OR “human immuno-deficiency virus” OR “human immune-deficiency virus” OR “acquired immunodeficiency syndrome” OR “acquired immunedeficiency syndrome” OR “acquired immunodeficiency syndrome” OR “acquired immune-deficiency syndrome” OR “hiv-positive” OR “sexually transmitted diseases” | 117,637 |
| S2 | (MH “mortality”) OR (MH “morbidity”) OR (MH "AIDS-Related Opportunistic Infections") OR “opportunistic infection*” OR OR “survival status” OR “death rate” OR “case fatality rate” OR “mortality determina*” OR “mortality rat*” OR “mortality predict*” OR “risk of mortality” OR “morbidit*” OR “hospital admissions” OR “hospitalization” OR “herpes zoster” OR “bacterial pneumonia” OR “pulmonary TB” OR “extra-pulmonary TB” OR “oral candidiasis” OR “oesophageal candidiasis” OR “mouth ulcer” OR “diarrhoea” OR “pneumocystis pneumonia” OR “central nervous system toxoplasmosis” OR “cryptococcal meningitis” OR “non-Hodgkins lymphoma” OR “Kaposi’s sarcoma” OR “cervical cancer” OR “herpes simplex” OR “cytomegalovirus” | 226,082 |
| S1 | (MH "Malnutrition") OR (MH “Body Mass Index”) OR “undernutrition” OR “malnourishmt*” OR “nutritional deficienc*” OR “nutritional status” OR “BMI” OR “micronutrient deficienc*” OR “stunting” “wasting” OR “underweight” | 117,352 |
